# Meta-Analysis: Prevalence of Youth Mental Disorders in Sub-Saharan Africa

**DOI:** 10.1017/gmh.2024.82

**Published:** 2024-11-14

**Authors:** Cecilia E. Jakobsson, Natalie E. Johnson, Brenda Ochuku, Rosine Baseke, Evelyn Wong, Christine W. Musyimi, David M. Ndetei, Katherine E. Venturo-Conerly

**Affiliations:** 1 Shamiri Institute, Nairobi, Kenya; 2 Division of Clinical Epidemiology, Department of Clinical Research, University Hospital Basel, Basel, Switzerland; 3 School of Medicine, Stanford University, Stanford, CA, USA; 4 Africa Mental Health Research and Training Foundation, Nairobi, Kenya; 5 Department of Psychiatry, University of Nairobi, Nairobi, Kenya; 6 World Psychiatric Association Collaborating Centre for Research and Training, Nairobi, Kenya; 7 Department of Psychology, Harvard University, Cambridge, MA, USA

**Keywords:** sub-Saharan Africa, youth, mental health, prevalence, child and adolescent

## Abstract

Youth in sub-Saharan Africa (SSA) face limited access to professional mental health resources. A comprehensive assessment of the prevalence of mental disorders would build an understanding of the scope of the need.

We conducted systematic searches in PsycInfo, Pubmed, AfriBib and Africa Journals Online to identify prevalence rates for five disorders (anxiety, depression, conduct disorder, attention problems and post-traumatic stress) among SSA youth with a mean age of less than 19 years. We calculated a random-effects pooled prevalence for each disorder and assessed possible moderators.

The meta-analysis included 63 studies with 55,071 participants. We found the following pooled prevalence rates: 12.53% post-traumatic stress disorder (PTSD), 15.27% depression, 6.55% attention-deficit hyperactivity disorder, 11.78% anxiety and 9.76% conduct disorder. We found high heterogeneity across the studies, which may have resulted from differences in samples or measurement tools. Reported prevalence rates were not explained by the sample (i.e., special or general population), but whether the psychometric tool was validated for SSA youth affected the reported prevalence of PTSD and anxiety. In a meta-regression, prevalence rates were associated with the disorder type, with a higher prevalence of depression and PTSD. We found the mean age significantly moderated the prevalence in univariate meta-regression, with increased age correlated with greater prevalence.

Our findings suggest there is a need to explore reasons for varying prevalence rates further and to develop interventions that support youth mental health in SSA, particularly interventions for depression and PTSD. Limitations included a lack of standardization in psychometric tools and limited reporting on research methods, which influenced quality rating. Importantly, the search only considered studies published in English and was conducted 2 years ago. Although recent estimates reported slightly higher than our prevalence estimates, these reviews together highlight the prevalence and importance of youth mental health difficulties in SSA.

## Impact statement

The synthesis of 63 articles in this study gives a glimpse into the prevalence of five common psychiatric conditions: conduct disorder, depression, anxiety, attention-deficit hyperactivity disorder and post-traumatic stress disorder among sub-Saharan African youth. The high rates of depression, post-traumatic stress disorder and anxiety underscore the urgency for targeted interventions and policy reform. Our review compares the prevalence of these conditions among sub-Saharan African youth to global estimates for these conditions. It also calls attention to the pressing need for culturally sensitive and standardized assessments to measure mental health conditions.

## Introduction

An estimated 13% of all adolescents have at least one diagnosed mental disorder (Kuehn, 2021; UNICEF, 2021). In sub-Saharan Africa (SSA), 23% of the population (256 million people) is between the ages of 10–19 years (UNICEF, 2014; Agyepong et al., 2017; Sequeira et al., 2022) and SSA adolescents are the fastest-growing population in the world (Sequeira et al., 2022). Additionally, despite carrying a vast proportion of the global burden of mental disorders, the ratio of psychiatrists to the population of most SSA countries sits at less than 1 per 1,000,000 (WHO, 2021a), and the ratio of child psychiatrists is even lower (at 1 per 4,000,000 people) (Belfe and Saxena, 2006).

A previous meta-analysis estimated that 26.9% of youths aged 10–19 years in SSA experienced depression, 29.8% reported anxiety, 40.8% emotional and behavioural problems, 21.5% for post-traumatic stress disorder (PTSD) and 20.8% suicidal ideation (Jörns-Presentati et al., 2021). The high prevalence of mental disorders suggests that research is needed to support the availability and accessibility of mental health interventions to promote well-being (Cortina et al., 2012; Jörns-Presentati et al., 2021). Another meta-analysis estimated that 14.3% of children aged 0–16 years in SSA had at least one mental disorder (Cortina et al., 2012). As these studies present the data differently, with some focusing on specific disorders (e.g., depression and anxiety) and some on psychopathology in general, it is difficult to draw overall prevalence rates of mental disorders among youths in SSA. While previous reviews have assessed the prevalence of mental disorders among SSA youth (Jörns-Presentati et al., 2021), to our knowledge, none so far have assessed examined factors that may moderate the prevalence of mental disorders among SSA youth (e.g., use of diagnostic vs. screening procedures, special vs. general population), which is an important step forward to address challenges in mental healthcare for SSA youth.

Consolidating prevalence data on mental disorders among youth in SSA may be useful not only for better understanding the epidemiology of mental disorders in SSA but also for considering what resources would be most beneficial in this setting. To add to previous work analyzing the overall prevalence of general psychopathology in SSA, our meta-analysis was conducted with the aim of determining the prevalence of multiple common mental disorders in SSA; this work may be a step towards developing targeted interventions and supporting appropriate mental health policies. We evaluated whether reported prevalence rates were affected by the disorder type, year of study, mean age of the sample, percentage of female participants, type of psychometric scale (i.e., diagnostic or screening), whether the scale was validated in context, special population status (e.g., former child soldiers, trauma survivors) and region (East, West, Central or Southern Africa) (African Development Bank, 2018).

## Method

The study was pre-registered on PROSPERO CRD42022326574 (see Supplement 1) and conducted in accordance with PRISMA guidelines (see Supplement 2) (Page et al., 2021). The search strategy was developed by three researchers with support from a university librarian. The disorders selected are the five most identified mental health conditions among youth (i.e., depression, PTSD, anxiety, conduct disorder and attention-deficit hyperactivity disorder [ADHD]) (Weisz and Kazdin, 2017). To assess the effectiveness of the search strategy, the search terms were trialled in the various databases, and the researchers made adaptations to ensure that the search yielded relevant studies (see Appendix A). The three researchers also created and piloted the eligibility criteria to identify appropriate studies.

The systematic search was conducted on 20 December 2021 using the following four databases: PsycInfo (*n* = 928), Pubmed (*n* = 2,858), AfriBib (*n* = 234) and Africa Journals Online (*n* = 100 articles could be accessed). The available articles from all four databases were exported to EndNote X9 (EndNote Team, 2013) and uploaded to Rayyan (Ouzzani et al., 2016), where duplicate records were identified and removed.

### Screening

The title, abstract and full text of each search result were independently double-screened by four authors using the piloted pre-specified inclusion criteria. The inclusion criteria included the following: (1) an empirical study, (2) published in English (as this was the only language that all authors spoke fluently), (3) involving participants from SSA with a mean age of less than 19 years, (Viner, 2013) and (4) including a prevalence measure of one or more of the selected disorder types (i.e., anxiety problems, depression problems, conduct problems, ADHD and post-traumatic stress disorder [PTSD]). We defined a prevalence measure as a measurement taken with a tool used to identify a mental health diagnostic status or an established (i.e., psychometrically validated in some setting) measure of symptom levels. The studies could be of a general or special population (e.g., youth living with HIV). Studies were excluded if the participants were already selected or self-selected for the presence of mental disorders or symptoms (i.e., people already seeking or receiving mental health care). Furthermore, if several mental disorders were included, the studies needed to report a prevalence for each condition (i.e., not a general measure for distress or disorder). Additionally, we excluded studies using non-probabilistic search strategies to mitigate the risk of bias in prevalence estimates (see Appendix A).

### Data Extraction

Data pertaining to the following were extracted from each included article: (a) study characteristics, (b) participant characteristics and (c) prevalence of included mental disorder(s). Four researchers completed the data extraction independently; however, any ambiguity in reporting was explored through weekly meetings. The characteristics extracted from each study included study location by country, objectives of the study and study design. The extracted participant and study characteristics included: sample size, age range, mean age, percent female, sampling method and, if applicable, the special population characteristics (e.g., juvenile offenders).

Regarding the prevalence of selected mental disorders, the following information was extracted: selected disorder, psychometric scale(s) used, informant (i.e., self-reported, teacher- or parent-reported) and prevalence measure. For studies that included participants from multiple regions or reported prevalence rates for more than one of the five psychiatric disorders, we extracted the sample size, mean age, percentage of female participants and psychometric scale type as reported for each disorder and/or region. We also investigated whether the psychometric scales were culturally validated in the study’s context. For manuscripts that reported that the chosen tools were validated, we assumed they were indeed validated. For manuscripts that did not include information about scale validation, we cross-checked the broader literature to determine whether the scales were validated at the time of their inclusion in the studies (indicated as “No” if the scale was not validated, “Yes” if the scale was validated, or “Yes†” if the scale had been validated after the study).

### Quality Appraisal

Each study was evaluated by two independent authors using the Johanna Briggs Institute Tools for cohort and cross-sectional study designs (Moola et al., 2015). Any discrepancies in the appraisals were resolved by consensus.

### Data Analysis

Prevalence rates were obtained from each included study and organized by selected disorder, as presented in Table 2. When studies included more than one prevalence rate for the same disorder (e.g., multiple scales used to assess the same condition), a weighted average of all reported prevalence rates was calculated by two authors. For studies that did not provide a confidence interval (CI) around the prevalence estimate, the 95% CIs for all reported prevalence rates were calculated (Eberly College of Science, 2022).

Using the *metafor* package in R (Version 4.3.1 (2023-06-16)) (Viechtbauer, 2022), logit-transformed proportions were calculated for each prevalence rate, and the inverse variance method was applied to estimate the pooled prevalence of each condition (Berkey et al., 1998; Harrer et al., 2021). We then used a mixed-effects meta-regression, with the proportion specified as a random effect and the sub-group variable specified as a fixed effect, and a logit-transformation applied to the proportion to test the following hypothesized moderators: year of study, location, psychometric scale type, disorder type, mean age of the sample, percentage of female participants, region or special population status.

To assess the heterogeneity of the studies included in the review, forest plots were created for each of the five specified disorders. Furthermore, sensitivity analyses were conducted to assess heterogeneity (*I*^2^) after the removal of studies of a special population or those that used a non-validated tool. Sensitivity analyses were also employed to evaluate heterogeneity after the removal of outliers and influential cases that were identified through influence (Viechtbauer and Cheung, 2010) and Graphic Display of Heterogeneity (GOSH) plot (Olkin et al., 2012) diagnostics. Finally, subgroup analyses were conducted to investigate the variance in prevalence between general and special populations for culturally validated measures compared to non-validated measures and for studies that used a screening tool compared to those that used a diagnostic tool.

## Results

The systematic search identified 4,120 search hits, with 3,783 studies included in screening after removing duplicates. After the title and abstract screening process, 3,639 studies were excluded and 140 studies underwent full-text screening. As a result, 77 more studies were excluded, and 63 studies were included in the final review (see Supplement 2).

As seen in Table 1, the studies included in this meta-analysis (*n* = 63) were conducted in 14 countries across SSA. The most common locations were Nigeria (*n* = 15), Kenya (*n* = 12) and South Africa (*n* = 10). The remaining studies were conducted in Uganda (*n* = 8), Ethiopia (*n* = 4), Tanzania (*n* = 3), Ghana (*n* = 3), Democratic Republic of Congo (*n* = 2), Rwanda (*n* = 1), Malawi (*n* = 1), Burundi (*n* = 1), Namibia (*n* = 1), Zambia (*n* = 1) and Zimbabwe (*n* = 1). Most studies were cross-sectional (*n* = 57), and six studies employed a cohort design. The studies included in this review varied in sample sizes, populations and sampling methods. Sample sizes ranged from 31 participants to 4,795 participants. The total sample size of all included studies was 55,071. The most common sampling method was cluster sampling (*n* = 24). Other studies utilized stratified random (*n* = 8), multistage (*n* = 7), random (*n* = 6), systematic (*n* = 3) and population-based (*n* = 2) sampling. Participant ages ranged from 0 to 28 years, and the mean age of the total sample was 13.63 (SD = 2.52). We included the mean or median age, as reported in the studies. Overall, the total sample comprised 46.65% females. As seen in Table 2, more than half of the studies reported on the prevalence of mental disorders in the general population, 30 articles studied a special population as follows: HIV-positive children/adolescents (*n* = 11), violence-affected youth (*s* = 3), refugees (*n* = 2), orphans (*n* = 2), juvenile offenders (*n* = 2), children seeking primary medical care (*n* = 2), rape survivors (*n* = 1), former child soldiers (*n* = 1), child labourers (*n* = 1), cancer patients (*n* = 1), pregnant adolescents (*n* = 1), youth in vocational training (*n* = 1) and overweight and obese children (*n* = 1). Most studies included in this review assessed depression (*n* = 34), followed by ADHD (*n* = 23), anxiety disorders (*n* = 22), PTSD (*n* = 19) and conduct problems (*n* = 12). The disorders were measured using different informants, including caregivers (*n* = 20), teachers (*n* = 11) and self-reports (*n* = 45) (see Table 2). Furthermore, in the 63 studies, six tools were not culturally validated, and nine scales were validated in the context after the study was published. Among the 63 included studies, 38 unique scales were used; of these, 13 were diagnostic, and 25 were screening tools. Most (*n* = 20) of the scales were only used in one study. The most frequently used psychometric tools were the Disruptive Behaviour Disorder Rating Scale (DBDRS) for ADHD (*n* = 6), Mini International Neuropsychiatric Interview for Children and Adolescents (MINI-KID) for depression (*n* = 6), Patient Health Questionnaire-9-item (PHQ-9) for depression (*n* = 8) and the Children’s Depression Inventory (CDI) for depression (*n* = 6). See Appendix C for additional details on the scales used.

**Table 1.** Study characteristics

Reference	Country	Study design	Sampling method	Sample size	Min age (years)	Max age	Mean age	SD	Percent female	Population
Crombach et al. (2014)	Burundi	Cross-sectional	Random	112	10	23	15.9	3.1	0	Homeless children
Ndukuba et al. (2017)	Nigeria	Cross-sectional	Random	181	NR	NR	9.39	1.97	46.4	General
Roberts et al. (2022)	South Africa	Cohort	Multistage	723	13	18	15‡	NR	100	HIV+
Kashala et al. (2005)	Congo	Cross-sectional	Stepwise cluster	1187	NR	NR	8.33	9	46	General
Zeegers et al. (2010)	South Africa	Cross-sectional	Cluster	100	NR	NR	8‡	NR	49	HIV+
Adewuya and Famuyiwa (2007)	Nigeria	Cross-sectional	Multistage	1112	NR	NR	8.94	2.1	38.7	General
Chinawa et al. (2014)	Nigeria	Cross-sectional	Cluster	273	2	13	7.06	NR	52.5	General
Rukabyarwema et al. (2019)	Rwanda	Cross-sectional	Cluster	235	6	16	10.1	NR	44	Children seeking primary medical care
Kariuki et al. (2017)	Kenya	Cross-sectional	Random	3273	NR	NR	4‡	NR	49	General
Cortina et al. (2013)	South Africa	Cross-sectional	Stratified random	1025	10	12	NR	NR	49.2	Refugees
Ambuabunos et al. (2011)	Nigeria	Cross-sectional	Multistage random	1473	NR	NR	9.3	2	46.8	General
Tirfeneh and Srahbzu (2020)	Ethiopia	Cross-sectional	Simple random	624	15	16	NR	NR	54.3	General
Girma et al. (2021)	Ethiopia	Cross-sectional	Multistage simple random	546	14	19	16.83	1.3	60.3	General
Osok et al. (2018)	Kenya	Cross-sectional	Cluster	176	15	18	NR	NR	100	Pregnant adolescents
Bukenya et al. (2022)	Uganda	Cross-sectional	Cluster	305	13	25	17.9	2.3	82.3	Youth in vocational training
Teivaanmäki et al. (2018)	Malawi	Cross-sectional	Cluster	523	NR	NR	15	NR	51	General
Mutiso et al. (2017)	Kenya	Cross-sectional	Stratified random	630	10	18	NR	NR	54.9	Orphans
Dow et al. (2016)	Tanzania	Cross-sectional	Cluster	182	NR	NR	17.2	2.9	54	HIV+
Barhafumwa et al. (2016)	South Africa	Cross-sectional	Stratified random	789	NR	NR	18‡	NR	58	General
Oshodi et al. (2020)	South Africa	Cohort	Cluster	31	14	18	15.4	1.2	100	Rape survivors
Atwoli et al. (2014)	Kenya	Cross-sectional	Stratified random	1451	NR	NR	13.8	2.2	44.5	Orphans
Ashaba et al. (2018)	Uganda	Cross-sectional	Cluster	224	13	17	14	NR	58	HIV+
Kinyanda et al. (2019)	Uganda	Cohort	Cluster random	1336	5	17	NR	NR	52.2	HIV+
Haas et al. (2020)	South Africa	Cohort	Cluster	1088	10	19	NR	NR	49.5	HIV+
West et al. (2019)	South Africa	Cross-sectional	Cluster	278	9	19	NR	NR	53	HIV+
Nkuba et al. (2018)	Tanzania	Cross-sectional	Random	700	NR	NR	14.92	1.02	52	General
Imasiku and Banda (2010)	Zambia	Cross-sectional	Cluster	74	7	17	14	2.69	8.1	Homeless children
Gureje et al. (1994)	Nigeria	Cross-sectional	Cluster	990	7	14	9.3	2.2	NR	Children seeking primary medical care
Harder et al. (2012)	Kenya	Cohort	Random	552	2	18	11	NR	52	Violence-affected youth
Haney et al. (2014)	Zimbabwe	Cross-sectional	Population-based survey	4795	15	24	18.8	NR	54.9	General
Maru et al. (2003)	Kenya	Cross-sectional	Cluster	90	8	18	NR	NR	28.9	Juvenile offenders
Kamau et al. (2012)	Kenya	Cross-sectional	Cluster	162	6	18	9.7	2.8	48.1	HIV+
Adefalu et al. (2018)	Nigeria	Cross-sectional	Cluster	196	6	17	9.2	NR	52.6	HIV+
Okwaraji et al. (2017)	Nigeria	Cross-sectional	Simple random	560	14	22	16.72	1.93	50	Violence-affected youths
Oke et al. (2019)	Nigeria	Cohort	Multistage random	1385	5	12	8.3	2.1	50.4	General
Atilola et al. (2014)	Nigeria	Cross-sectional	Cluster	288	15	19	18.5	1.5	0	Juvenile offenders
Peltzer (1999)	South Africa	Cross-sectional	Multistage	148	NR	NR	12.1	3.1	54	General
Scharpf et al. (2019)	Tanzania	Cross-sectional	Random	230	7	15	12.11	2.03	47.4	Refugees
Mpango et al. (2017)	Uganda	Cross-sectional	Cluster	1339	5	17	NR	NR	52	HIV+
Umar et al. (2018)	Nigeria	Cross-sectional	Multistage	487	NR	NR	14.09	1.85	48.5	General
Fatiregun and Kumapayi (2014)	Nigeria	Cross-sectional	Stratified cluster	1713	0	19	13.96	2.08	55.3	General
Afeti and Nyarko (2017)	Ghana	Cross-sectional	Stratified random	400	2	12	9.6	NR	42.7	General
Nalugya-Sserunjogi et al. (2016)	Uganda	Cross-sectional	Stratified random	519	14	16	16	2.18	42	General
Akimana et al. (2019)	Uganda	Cross-sectional	Cluster	352	7	17	11.5	3.2	31.82	Cancer patients
Kusi-Mensah et al. (2019)	Ghana	Cross-sectional	Cluster	303	7	15	9.22	NR	48.5	General
Rochat et al. (2018)	South Africa	Cross-sectional	Population-based survey	1,534	7	11	NR	NR	NR	General
Abbo et al. (2013)	Uganda	Cross-sectional	Multistage	1,587	3	19	NR	NR	NR	General
Adewuya and Ola (2005)	Nigeria	Cross-sectional	Cluster	102	12	18	14.46	1.98	36.28	General
Ashenafi (2001)	Ethiopia	Cross-sectional	Systematic	1,447	5	15	NR	NR	49.4	General
Okewole et al. (2015)	Nigeria	Cross-sectional	Cluster	700	12	19	15.4	1.3	23	General
Mels et al. (2009)	Democratic Republic of Congo	Cross-sectional	Cluster	1,046	13	21	15.8	1.8	45.6	General
Ruiz-Casares et al. (2009)	Namibia	Cross-sectional	Systematic	157	8	21	14.9	3.1	51	General
Ertl et al. (2014)	Uganda	Cross-sectional	Multistage cluster	1,113	12	25	18.79	3.79	22.27	Former child soldiers, violence-affected youths
Ndetei et al. (2008)	Kenya	Cross-sectional	Stratified	3,775	13	28	NR	NR	40.82	General
Khasakhala et al. (2012)	Kenya	Cross-sectional	Stratified random	1,276	13	22	NR	NR	42.2	General
Ndetei et al. (2016b)	Kenya	Cross-sectional	Simple random	2,267	10	12	NR	NR	51.9	General
Fekadu et al. (2006)	Ethiopia	Cross-sectional	Systematic	1,000	5	15	NR	NR	56	Child laborers
Mbwayo et al. (2020)	Kenya	Cross-sectional	Stratified random	2,482	11	17	NR	NR	50	General
Swain et al. (2017)	South Africa	Cross-sectional	Stratified	320	9	18	13.11	NR	65	General
Gaitho et al. (2018)	Kenya	Cross-sectional	Cluster	270	10	19	14.75	NR	46.3	HIV+
Anokye et al. (2020)	Ghana	Cross-sectional	Multistage	1,540	5	13	9	2.16	48	General
Adewuya et al. (2007)	Nigeria	Cross-sectional	Multistage	1,095	13	18	15.25	1.68	42.3	General
Okeke et al. (2018)	Nigeria	Cross-sectional	Multistage stratified random	200	10	18	12.9	1.8	28.0	Overweight and obese children

*Note:*

^†^ *Weighted average, excluding studies that did not report these figures;*

^‡^ *Median ag*e

**Table 2.** Prevalence data

Reference	Selected disorder	Baseline prevalence* (%)	95% Confidence interval (%)	Informant	Psychometric scales*‡*	Type of scale	Validated in context
Crombach et al. (2014)	PTSD Major depression	20.5 6.1	13.0–28.0 1.7–10.5	Self	UCLA PTSDRI MINI-KID	Screening diagnosis	Yes Yes
Ndukuba et al. (2017)	ADHD	6.6	3.0–10.2	Teacher	ARS-IV	Diagnosis	No
Roberts et al. (2022)	Depression Anxiety PTSD	6.9 1.4 0.6	5.1–8.7 0.5–2.3 5.1–8.7	Self	CDI-S RCMAS Child PTSD Checklist	Screening Diagnosis screening	Yes Yes Yes
Kashala et al. (2005)	ADHD	5.9	4.6–7.2	Caregiver, teacher	DBDRS	Screening	Yes*†*
Zeegers et al. (2010)	ADHD	16.9	9.6–24.2	Caregiver, teacher	SNAP-IV	Screening	Yes*†*
Adewuya and Famuyiwa (2007)	ADHD Conduct problems anxiety/ Depression	8.7 9.3 20.6	7.0–10.4 7.6–11.0 18.2–23.0	Caregiver, teacher	VADTRS VADPRS	Diagnosis Diagnosis	No No
Chinawa et al. (2014)	ADHD	3.2	1.1–5.3	Self	DBDRS	Screening	Yes*†*
Rukabyarwema et al. (2019)	ADHD, Anxiety/ Depression Conduct Problems	13.0 20.0 11.0	8.7–17.3 14.9–25.1 7.0–15.0	Caregiver	PSC	Screening	Yes
Kariuki et al. (2017)	Anxiety Anxiety/ Depression ADHD	12.6 12.7 5.0	11.5–13.7 11.6–3.8 4.3–5.7	Caregiver	CBCL	Screening	Yes
Cortina et al. (2013)	PTSD Anxiety/ Depression	23.9 14.1	21.3–26.5 12.0–16.2	Teacher, self	TSCC-A YSR	Screening Screening	Yes Yes
Ambuabunos et al. (2011)	ADHD	7.6	6.2–9.0	Caregiver, teacher	DBDRS	Screening	Yes*†*
Tirfeneh and Srahbzu (2020)	Depression	36.2	32.4–40.0	Self	PHQ–9	Screening	Yes
Girma et al. (2021)	Depression	28.0	24.2–31.8	Self	PHQ–9	Screening	Yes
Osok et al. (2018)	Depression	33.0	26.1–39.9	Self	PHQ–9	Screening	Yes
Bukenya et al. (2022)	Depression PTSD Anxiety	49.8 53.8 34.4	44.2–55.4 48.2–59.4 29.1–39.7	Self	PHQ–9 PC-PTSD GAD–7	Screening Screening Screening	Yes No Yes
Teivaanmäki et al. (2018)	Depression	90.0	87.4–92.6	Self	SMFQ	Screening	No
Mutiso et al. (2017)	Conduct problems	5.1	3.4–6.8	Self	YSR	Screening	Yes
Dow et al. (2016)	Depression PTSD	12.1 10.4	7.4–16.8 6.0–14.8	Self	PHQ–9 UCLA-PTSDRI	Screening Screening	Yes
Barhafumwa et al. (2016)	Depression	33.0	29.7–36.3	Self	CES-DC	Screening	Yes
Oshodi et al. (2020)	Depression Anxiety PTSD	22.6 16.1 12.9	7.9–37.3 3.2–29.0 1.1–24.7	Self	MINI-KID	Diagnosis	Yes
Atwoli et al. (2014)	PTSD	13.9	12.1–15.7	Self	Child PTSD checklist	Screening	Yes
Ashaba et al. (2018)	Depression	16.0	11.2–20.8	Self	MINI-KID	Diagnosis	Yes
Kinyanda et al. (2019)	Depression	5.0	3.8–6.2	Self	CASI–5	Diagnosis	Yes
Haas et al. (2020)	Depression Anxiety PTSD	15.5 15.1 0.7	13.3–17.7 13.0–17.2 0.2–1.2	Self	PHQ–9 GAD–7 PC-PTSD–5	Screening Screening Screening	Yes Yes Yes*†*
West et al. (2019)	Depression Anxiety PTSD	7.6 6.7 5.3	4.5–10.7 3.8–9.6 2.7–7.9	Self	CDI RCMAS Child PTSD checklist	Screening Diagnosis Screening	Yes Yes Yes
Nkuba et al. (2018)	Conduct problems ADHD	41.5 16.6	37.2–45.8 13.4–19.8	Caregiver, self	SDQ	Screening	Yes
Imasiku and Banda (2010)	Conduct problems ADHD	24.8 3.7	15.0–34.6 −0.6–8.0	Caregiver, self	SDQ	Screening	Yes*†*
Gureje et al. (1994)	Conduct problems Depression Anxiety ADHD	6.1 6.0 4.7 1.1	4.6–7.6 4.5–7.5 3.4–6.0 0.5–1.7	Self	DISC K-SADS-PL	Diagnosis Diagnosis	Yes*†* Yes
Harder et al. (2012)	PTSD	18.0	14.8–21.2	Caregiver, self	UCLA PTSDRI	Screening	Yes
Haney et al. (2014)	Depression	3.5	3.0–4.0	Self	SRQ–20	Screening	Yes
Maru et al. (2003)	Conduct problems	20.0	11.7–28.3	Self	RQC	Screening	Yes
Kamau et al. (2012)	Anxiety Depression ADHD	32.2 17.8 12.2	25.0–39.4 11.9–23.7 7.2–17.2	Self	MINI-KID	Diagnosis	Yes
Adefalu et al. (2018)	ADHD	4.6	1.7–7.5	Caregiver	CBQ	Screening	No
Okwaraji et al. (2017)	PTSD Depression Anxiety	24.3 7.9 11.4	20.7–27.9 5.6–10.0 8.8–14.0	Self	Short screening scale for PTSD BDI-II GAD–7	Screening Screening Screening	Yes Yes Yes
Oke et al. (2019)	ADHD	4.7	3.6–5.8	Caregiver, teacher	DBDRS	Screening	Yes
Atilola et al. (2014)	PTSD	5.8	3.1–8.5	Self	K-SADS-PL	Diagnosis	Yes
Peltzer (1999)	PTSD	8.4	3.9–12.9	Self	Children’s PTSD Inventory	Diagnosis	Yes
Scharpf et al. (2019)	PTSD	5.7	2.7–8.7	Self	UCLA PTSDRI	Screening	Yes
Mpango et al. (2017)	ADHD	6.0	4.7–7.3	Caregiver, teacher	CASI–5	Diagnosis	Yes
Umar et al. (2018)	ADHD	8.8	6.3–11.3	Self	K-SADS-PL	Diagnosis	Yes
Fatiregun and Kumapayi (2014)	Depression	21.2	19.3–23.1	Self	PHQ–9	Screening	Yes
Afeti and Nyarko (2017)	ADHD	12.75	9.5–16.0	Caregiver, teacher	DBDRS	Screening	Yes*†*
Nalugya-Sserunjogi et al. (2016)	Depression	21.0	17.5–24.5	Self	CDI	Screening	Yes
Akimana et al. (2019)	Depression	26.0	21.4–30.6	Self	MINI-KID	Diagnosis	Yes
Kusi-Mensah et al. (2019)	Depression Anxiety ADHD Conduct problems	1.3 1.0 1.6 2.0	0.0–2.6 −0.1–2.1 0.2–3.1 0.4–3.5	Caregiver, teacher	K-SADS-PL	Diagnosis	Yes
Rochat et al. (2018)	Conduct problems ADHD Anxiety	11.8 4.4 5.0	10.2–13.4 3.4–5.4 3.9–6.1	Caregiver	CBCL	Screening	Yes
Abbo et al. (2013)	Anxiety PTSD	26.6 6.6	24.4–28.8 5.4–7.8	Caregiver, teacher	MINI-KID	Diagnosis	Yes
Adewuya and Ola (2005)	Anxiety Depression	31.4 28.4	22.4–40.4 19.7–37.2	Caregiver	DISC-IV	Diagnosis	Yes
Ashenafi (2001)	ADHD Anxiety Depression	1.5 1.6 0.9	0.9–2.1 1.0–2.2 0.4–1.4	Caregiver	DICA	Diagnosis	Yes
Okewole et al. (2015)	Conduct problems ADHD	16.7 8.9	13.9–19.5 6.8–11.0	Self	SDQ	Screening	Yes
Mels et al. (2009)	PTSD	52.2	49.2–55.2	Self	IES-R	Screening	Yes
Ruiz-Casares et al. (2009)	Depression	1.9	−0.2–4.0	Self	CDI	Screening	Yes
Ertl et al. (2014)	PTSD	15.0	12.9–17.1	Self	PDS	Diagnosis	Yes
Ndetei et al. (2008)	Depression Anxiety	43.7 12.9	42.1–45.3 11.8–14.0	Self	MASC CDI NOK	Screening Screening Screening	Yes Yes Yes
Khasakhala et al. (2012)	Depression	26.4	24.0–28.8	Self	CDI	Screening	Yes
Ndetei et al. (2016b)	Conduct Problems ADHD Anxiety	12.5 29.6 8.2	11.1–13.9 27.7–31.5 7.1–9.3	Self	YSR	Screening	Yes
Fekadu et al. (2006)	Anxiety Conduct Problems	4.8 1	3.5–6.1 0.4–1.6	Self	DICA	Diagnosis	Yes
Mbwayo et al. (2020)	PTSD	26.8	25.0–28.6	Self	UCLA PTSDRI	Screening	Yes
Swain et al. (2017)	PTSD	5.9	3.4–8.5	Caregiver	TSCC	Screening	Yes
Gaitho et al. (2018)	Depression	52.6	46.6–58.6	Self	PHQ–9	Screening	Yes
Anokye et al. (2020)	ADHD	5.0	3.9–6.1	Caregiver	DBDRS	Screening	Yes*†*
Adewuya et al. (2007)	Major depression	6.90	5.4–8.4	Self	K-SADS-E	Diagnosis	Yes
Okeke et al. (2018)	Depression Anxiety	46.0 14.0	39.1–52.9 9.2–18.8	Self	BDI-II RCMAS	Screening Diagnosis	Yes

Note: *Above are the prevalence from each study, accompanied by the psychometric tools and their contextual validation indicated as “No” if the scale was not validated, “Yes” if the scale was validated, or “Yes†” if the scale had been validated after the study. Additional details of psychometric scales can be found in Appendix C.*

Based on the prevalence reported in Table 2, we calculated the following pooled prevalence rates for youth mental health conditions: depression 15.27% [CI 9.92; 22.78], PTSD 12.53% [CI 7.59; 20.00], anxiety disorders 11.78% [CI 7.27; 18.54], conduct disorder 9.76% [CI 4.93; 18.41] and ADHD 6.55% [CI 4.61; 9.23]. Additionally, there were two studies, Kamaue et al. (Kamau et al., 2012) and Gureje et al. (1994), that included estimates of several anxiety conditions in their studies. Gureje et al. (1994) detailed the following additional prevalence: separation anxiety 1.7% [CI 1.0–2.7], overanxious disorder 0.7% [CI 0.3–1.5], and simple phobia 2.0% [CI 1.2–3.1]. Similarly, Kamaue et al. (2012) listed panic disorder at 5.8%, agoraphobia at 2.6%, specific phobia at 7.1%, social phobia at 12.8%, panic disorders at 5.8% and separation anxiety disorder at 2.6%. The estimates of overall anxiety disorders from these studies were included in weighted average calculations, but we have listed these prevalence measures as additional information.

We observed high heterogeneity between studies for all conditions (see forest plots in Supplement 3). Sensitivity analyses indicated that heterogeneity did not substantially decrease after removing studies that included a special population, those that used a screening tool or tool that was not contextually validated. Similarly, we conducted a sensitivity analysis after removing outliers, studies with special population and non-validated tools and GOSH plot diagnostics did not substantially reduce heterogeneity (see Appendices D and F). Furthermore, we performed a subgroup analysis of population types, which revealed no significant differences between the pooled prevalence for studies that included a general vs. special population (see Appendix E). Subgroup analyses of validated vs. non-validated tools indicated a significant difference in the pooled prevalence of PTSD and anxiety. However, in both cases, only one study used a non-validated tool: Bukenya et al. (2022), which was conducted in Uganda using the PC-PTSD questionnaire, and Adewuya and Famuyiwa (2007), which was conducted in Nigeria using the VADTRS and VADPRS questionnaires. Because of the limited number of non-validated tools, these results must be interpreted cautiously. Finally, we carried out a subgroup analysis of psychometric tools, which demonstrated a significantly higher prevalence for depression and conduct disorder when a screening tool was used to estimate prevalence compared to a diagnostic tool (see Appendix G).

In the full model, the disorder type (i.e., ADHD, Anxiety, Depression, Conduct, PTSD) was found to be a significant predictor of prevalence, with depression and PTSD having a higher prevalence. Other than disorder type, none of the hypothesized moderators were significantly associated with an increased reported prevalence rate in the full model. However, given that previous studies have consistently reported differences in psychiatric morbidities across genders (Seedat et al., 2009; Remes et al., 2016; Van Droogenbroeck et al., 2018; Miranda-Mendizabal et al., 2019) and age (Park et al., 2014), we conducted an exploratory analysis to probe whether these variables were associated with increased reported prevalence rates when tested alone as individual moderators. When modelled separately, we found that the mean age of the participants significantly moderated the reported prevalence rates (*B =* 0.02, *p <* 0.001, *k* = 69). In contrast, the percentage of female participants did not significantly moderate the reported prevalence (*B =* 0.0009, *p =* 0.386, *k* = 100).

### Quality Appraisal

The studies ranged in the level of detail provided for the inclusion criteria and description of the study setting and participants, which may have impacted our evaluation of confounding variables. The average appraisal rating across the 57 cross-sectional studies included in this review was 6.74/8, indicating a moderate quality of the included cross-sectional studies. Regarding the six cohort studies included in this review, some did not detail their evaluation of confounding variables and loss to follow-up. As this information was uncertain, it was difficult to assess the overall quality of the studies, as it might have indicated possible bias. This resulted in a lower-quality rating for cohort studies included in this review, with an average rating of 6.83/11.

## Discussion

This meta-analysis evaluated the prevalence of five mental disorders among youth in SSA. The prevalence of depression was found to be the highest, at 15.27%, with PTSD having the second highest prevalence, at 12.53%. Anxiety had a prevalence rate of 11.78%, with conduct disorder observed among 9.76% and ADHD among 6.55% of SSA youth. This prevalence drew from cohort and cross-sectional findings across 14 countries in SSA involving a total sample of 55,07 participants with a mean age of 13.63 (SD = 2.52) (see Tables 1 and 2). The global estimates for depression and conduct disorder were greater than those found in this review (UNICEF, 2021; Shorey et al., 2022). The global prevalence of ADHD and anxiety were comparable to those calculated in this review (Merikangas et al., 2009; Thomas et al., 2015). Although there has not been a global estimate for PTSD among a general population, a previous meta-analysis of PTSD among trauma-affected youth found a similar prevalence to the one found by this review (Alisic et al., 2014). Additionally, a systematic review of PTSD prevalence in LMICs found a widely ranging prevalence of PTSD, similar to what has been found in this review (Yatham et al., 2018).

High between-study heterogeneity was observed, suggesting considerable variation in prevalence. Interestingly, heterogeneity did not substantially decrease despite removing outliers and influential cases. To further explore potential reasons for observed differences in reported prevalence rates, we assessed the significance of our hypothesized moderators as predictors in a meta-regression. We found that the disorder type significantly moderated the prevalence in the full model, and the mean age of the study sample significantly moderated reported prevalence rates when modelled separately, with increased age correlating to an increase in reported prevalence. The direction of this effect is consistent with other studies that indicated adolescence as a time of increased incidence of psychiatric morbidities, including mood disorders (Merikangas et al., 2009; WHO, 2021b; Sulley et al., 2022). However, these results are subject to ecological and aggregation biases and should be interpreted with caution. Our pooled prevalence combined a range of samples, with some being from a special population. When we conducted a subgroup analysis to determine if this impacted the resulting pooled prevalence rates, we found that for PTSD, ADHD and conduct disorder, the prevalence rate was lower among special populations (e.g., youth living with HIV, refugees, violence-affected youths, juvenile offenders) and for anxiety and depression, the rate was higher. However, these differences were not statistically significant (see Appendix E). Additionally, in a sensitivity analysis, when special populations were removed, heterogeneity did not decrease (see Appendix D).

SSA is a vast geographic region characterized by important cultural and contextual differences that may have impacted the diversity of results. Prior research suggests that mental disorder burdens are comparatively higher in East Africa (Cataldi, 2021; Ferrari et al., 2022), with less expenditure noted for neurological disorders in this region (Etindele Sosso and Kabore, 2016). However, while disorder type was significantly associated with a difference in reported prevalence rates, we found no significant effect for region nor the interaction between region and disorder type in our review.

Some of the included studies used several scales, including screening and diagnostic tools; however, typically, in the manuscripts of the included studies, only a single percentage was reported using results obtained via the screening tool. During data extraction, we indicated which type of scale (e.g., screening vs. diagnostic) was used to determine the included prevalence rate. We found screening tools were often used to indicate prevalence, which may be misleading as this approach cannot always differentiate between those at risk of a mental disorder and those who could be diagnosed with that disorder. Unfortunately, even in high-income countries with relatively well-staffed health systems, issues such as long waiting times for a formal diagnosis from a qualified practitioner often hinder the accurate tracking of diagnosed mental disorders (NHS, 2022). The subgroup analysis for diagnostic vs. screening tools found significant differences for depression and conduct disorder, where the pooled prevalence of studies using a diagnostic tool was lower than studies using a screening tool.

We also noted that most studies used self-report scales. However, the studies that included parent and teacher ratings often found higher rates of the given disorder than the self-reported scores, which aligns with previous studies that have shown discrepancies in inter-rater reports (Brown et al., 2006; Papageorgiou et al., 2008; Boman et al., 2016). Additionally, some authors used scales that had not been validated in the context of SSA. We found that when the scale was not validated in context, the rates of PTSD and anxiety were significantly higher (see Appendix F).

Overall, the studies were evaluated to be of moderate quality. There was a high level of uncertainty, with gaps in reporting such as consideration of confounding variables, reporting study/participant characteristics, and losing participants to follow-up, which impacted the level of quality in the assessed studies. The lack of reporting of some study quality indicators might suggest a need for more adequate reporting methods and analysis in these studies.

One limitation of this review is the lack of standardization in prevalence measurements. Depression, for example, was defined differently across studies, including depression problems, elevated depression symptoms, or major depressive disorder. Similarly, anxiety-related conditions were referred to as emotional problems, affective problems or anxiety disorders. Previous research has demonstrated the great clinical and diagnostic heterogeneity (Fried, 2017; Dennis-Tiwary et al., 2019; Athira et al., 2020; Drzewiecki and Fox, 2024) observed for both anxiety and depression. Thus, in the screening process, the authors of this meta-analysis considered the core symptoms described in each paper as well as the psychometric scales to ensure that these were screening or diagnostic tools for anxiety and depression. Still, the variety of measures used likely contributed to variation in the reported prevalence rates, thus increasing the confidence interval of the pooled prevalence. Relatedly, several scales were used to assess the prevalence of each disorder, which may have also contributed to the significant variation in the reported prevalence across studies. Furthermore, our review included 63 studies, six tools were not culturally validated, and nine scales were validated in the context after the study was published. Of the included studies, 38 unique scales were used; of these, 13 were diagnostic, and 25 were screening tools. Most (*n* = 20) of the scales were only used in one study (see Table 2).

Another limitation of this study is that the systemic search was conducted 2 years ago, and there have been several studies published since on the prevalence of youth mental health conditions. For example, Woolgar et al., 2022 found that preschool children were at risk of developing PTSD following exposure to trauma. They reported a pooled prevalence of 21.5% in their review and noted heterogeneity across the included studies as well as a lack of representation from LMICs. Another study (Yang et al., 2022) found an estimated global youth PTSD prevalence of 28.15% following the outbreak of coronavirus. Specific to youth in SSA, Jörns-Presentati et al., 2021 found a prevalence of 26.9% for depression, 29.8% for anxiety disorders, 40.8% for emotional and behavioural problems, 21.5% for PTSD and 20.8% for suicidal ideation. Another study on SSA youth (Hunduma et al., 2023) reported the following prevalence: 19% for depression, 20% for anxiety, 5% for ADHD and 15% for conduct disorders. Our meta-analysis included a larger sample of studies conducted in SSA and thus added to these previous studies. Although the estimates reported by recent reviewers are slightly higher than our prevalence estimates, these reviews together highlight the prevalence and importance of youth mental health difficulties in SSA.

The comparability of measures was not restricted to between studies only as, in some studies, more than one measure was used to assess the same disorder. Moreover, as reported in a previous meta-analysis (Cortina et al., 2012), there is a clear dearth of locally derived tools for measuring psychopathology. In this review, many researchers used measurement tools derived from Western populations to assess mental health symptoms. Although some studies reported on the psychometric properties of Western-derived tools when used in the SSA population, many did not. Furthermore, although psychometrically validated, some tools constructed in higher-income settings may not accurately capture culturally salient features of these disorders that are unique to the African population (Osborn et al., 2021). This indicates a need for more culture-sensitive, psychometrically validated tools tailored for use in the African population. It also indicates a need for standardized methods of assessing psychopathology. Relatedly, an important limitation of our study is that the search was conducted in English, and the inclusion of studies was limited to those published in English. Future meta-analyses and systematic reviews may include more languages to increase the representation of studies published in LMICs and SSA.

Our findings suggest a need to explore further reasons for the varying prevalence rates of studies across SSA. As our study demonstrated, there is currently an extensive range of psychometric tools used to assess mental disorders in this setting, which might lead to increased variation in prevalence estimates. Further research should find a way to reconcile multiple tools used to screen for and diagnose a single mental disorder to consolidate and compare rates of mental conditions across subgroups and populations. To scale up mental health interventions in SSA, we believe a closer investigation into the cultural and contextual factors—including socioeconomic variables, language and culture around mental health, and religious or spiritual beliefs—that may affect the prevalence of mental disorders would support the implementation process. Finally, although our review did not find a significant moderating effect of special populations, we believe that it would be necessary to evaluate these samples further to explore potential risk and protective factors for developing mental disorders within SSA. For example, there might be support systems that target at-risk youth, such as community-based organizations or non-governmental services, which may influence the prevalence rates.

Our meta-analysis revealed the differences in prevalence between types of mental disorders, which may implicate the clinical prioritization of certain conditions, such as PTSD and depression, in SSA. Despite the high prevalence rates of mental disorders in SSA, service availability remains limited. Previous research demonstrated that critical barriers to implementing youth mental health interventions include stigma, negative beliefs and having few delivery platforms outside of school-based settings, which may exclude individuals not attending formal education (Heflinger and Hinshaw, 2010; Jenkins et al., 2010; Ndetei et al., 2016a). The facilitators for implementing mental health interventions include positive experiences and mental health literacy; thus, positive psychology and psychoeducation may be priority mental health intervention research areas (Aguirre Velasco et al., 2020). Additionally, a recent meta-analysis has found that youth psychotherapies are particularly effective in LMICs compared to non-LMICs; thus, given the rates of mental disorders in LMICs combined with the promising effects of youth psychotherapies, researchers, funders and policy-makers may emphasize scale up of youth mental health interventions in SSA (Venturo-Conerly et al., 2023). Due to the high rates of mental disorders, our findings suggest that mental health care should be integrated into primary youth care, as early detection and intervention are critical for reducing the chronicity and severity of mental disorders. However, to support the successful integration of mental health into primary youth care, more research is needed to validate psychometric tools in local contexts. Furthermore, as recommended by Sequeira et al., 2022, a greater understanding of common mental health conditions and social determinates is needed to bridge the gap stigma creates.

## Supporting information

Jakobsson et al. supplementary material Jakobsson et al. supplementary material

## Data Availability

All articles included in this review are available in the described databases, and the sample characteristics and prevalence details are included in this article.

## Supplementary material

The supplementary material for this article can be found at http://doi.org/10.1017/gmh.2024.82.

## Appendices

### Appendix A: Search Strategies

PubMed

(((((("mental disorders"[MeSH Major Topic]) AND ((prevalence[Title/Abstract]) OR epidemiology OR incidence) AND ((("africa"[All Fields]) OR (sub-sahara*) OR (Algeria) OR (Angola) OR (Benin) OR (Botswana) OR (Burkina Faso) OR (Burundi) OR (Cabo Verde) OR (Cameroon) OR (Central African Republic) OR (Chad) OR (Comoros) OR (Congo) OR (Cote d’Ivoire) OR (Djibouti) OR (Egypt) OR (Equatorial Guinea) OR (Eritrea) OR (Eswatini) OR (Ethiopia) OR (Gabon) OR (Gambia) OR (Ghana) OR (Guinea) OR (Kenya) OR (Lesotho) OR (Liberia) OR (Libya) OR (Madagascar) OR (Malawi) OR (Mali) OR (Mauritania) OR (Mauritius) OR (Morocco) OR (Mozambique) OR (Namibia) OR (Niger) OR (Nigeria) OR (Rwanda) OR (Sao Tome and Principe) OR (Senegal) OR (Seychelles) OR (Sierra Leone) OR (Somalia) OR (South Africa) OR (South Sudan) OR (Sudan) OR (Tanzania) OR (Togo) OR (Tunisia) OR (Uganda) OR (Zambia) OR (Zimbabwe)))

Limits: Humans, Child (birth-18), Adolescent, Young-adult, abstract, full text

Africa Journal Online

mental (health OR illness OR disease OR disorder) AND (prevalence OR incidence) AND (young adult OR adolescent OR child)

AfriBib

Psychiatry

PsycInfo

(mental health OR mental illness OR mental disorder OR psychiatric illness OR behavioral disorders OR conduct disorders OR attention deficit hyperactivity disorder OR oppositional defiant disorder OR autism spectrum disorder OR anxiety OR depression OR Obsessive-Compulsive Disorder OR eating disorder OR bipolar OR schizophrenia? OR Post Traumatic Stress Disorder) AND (youth OR adolescent OR child* OR young adult) AND (Africa OR sub saharan OR (Algeria) OR (Angola) OR (Benin) OR (Botswana) OR (Burkina Faso) OR (Burundi) OR (Cabo Verde) OR (Cameroon) OR (Central African Republic) OR (Chad) OR (Comoros) OR (Congo) OR (Cote d’Ivoire) OR (Djibouti) OR (Egypt) OR (Equatorial Guinea) OR (Eritrea) OR (Eswatini) OR (Ethiopia) OR (Gabon) OR (Gambia) OR (Ghana) OR (Guinea) OR (Kenya) OR (Lesotho) OR (Liberia) OR (Libya) OR (Madagascar) OR (Malawi) OR (Mali) OR (Mauritania) OR (Mauritius) OR (Morocco) OR (Mozambique) OR (Namibia) OR (Niger) OR (Nigeria) OR (Rwanda) OR (Sao Tome and Principe) OR (Senegal) OR (Seychelles) OR (Sierra Leone) OR (Somalia) OR (South Africa) OR (South Sudan) OR (Sudan) OR (Tanzania) OR (Togo) OR (Tunisia) OR (Uganda) OR (Zambia) OR (Zimbabwe)) AND (prevalence OR epidemiology OR incidence)

### Appendix B. Eligibility Criteria for Included Studies

Pre-screening criteria:

1. **Youths**: The mean age is 0–19 years old. It can include studies that report a different mean age if the study reports results broken down by age and includes data on 0–18 years old.
2. **English**: The full article is available and published in English.
3. **SSA:** The study occurred in one or more countries in sub-Saharan Africa.
4. **Empirical article:** The article is an original empirical study (i.e., presents data collected for this study). Exclude meta-analyses, systematic reviews and narrative reviews.

Screening criteria:

1. **Prevalence measure:** Reports mental disorder prevalence using a measure of mental health diagnostic status or an established (i.e., psychometrically validated in some setting) measure of symptom levels.
  - Include studies that report the percentage of youth with a diagnosis or meeting a cutoff, and those that report raw numbers of those above a cutoff score on a scale.
  - If a study reports an odds ratio or prevalence ratio, but NO prevalence measure (i.e., % or raw number), do not include it. If a study reports an odds ratio and a prevalence measure of some kind, include it.
  - Do not include articles that only provide a mean score on a scale.
  - Do not include articles that only use one unvalidated item, or an unestablished collection of items, to measure the presence or absence of a disorder.
2. **Selected disorder**: Prevalence measure is of one or more of the following disorder types: anxiety problems (of all kinds, including OCD and specific phobias), depression problems, conduct problems, ADHD problems and post-traumatic stress disorder.
3. **Population**: The study is of the general population *or* a special population (e.g., youth with HIV, refugees) that is *not* already selected or self-selected for the presence of mental health problems or symptoms (i.e., the study cannot be of a group of people already seeking mental health services).
4. *Example exclusion*: Prevalence of anxiety and depression amongst a sample of youth seeking treatment at a mental health clinic.
5. *Example exclusion*: Prevalence of anxiety and depression amongst a sample of patients reporting post-traumatic disorder.
6. *Example inclusion*: Prevalence of post-traumatic stress disorder amongst children affected by conflict.
7. **Sampling strategy:** Researchers employed a probabilistic sampling strategy to recruit participants to their study.

### Appendix C. Psychometric Scale Used

**Table:**

Abbreviation	Psychometric scale	Mental health problem	Number of studies using this scale	Type of scale
ARS-IV	ADHD Rating Scale	ADHD	1	Diagnosis
BDI-II	Beck Depression Inventory	Depression	2	Screening
CASI–5	Child & Adolescent Symptom Inventory	Depression/ADHD	2	Diagnosis
CBCL	Child Behavior Checklist	Anxiety, anxiety/depression, ADHD	2	Screening
CBQ	Child Behavior Questionnaire	ADHD	1	Screening
CDI	Children’s Depression Inventory	Depression	5	Screening
CDI-S	Children’s Depression Inventory-Short	Depression	1	Screening
CES-DC	Center for Epidemiological Studies-Depression Scale for Children	Depression	1	Screening
Child PTSD Checklist	Child PTSD Checklist	PTSD	2	Screening
Children’s PTSD Inventory	Children’s PTSD Inventory	PTSD	1	Diagnosis
DBDRS	Disruptive Behavior Disorder Rating Scale	ADHD	6	Screening
DICA	Diagnostic Interview for Children & Adolescents	ADHD, anxiety, depression	2	Diagnosis
DISC	Diagnostic Interview Schedule for Children	Conduct problems, depression, anxiety, ADHD	1	Diagnosis
DISC-IV	Diagnostic Interview Schedule for Children—Version IV	Anxiety, depression	1	Diagnosis
GAD–7	Generalized Anxiety Disorder Assessment–7	Anxiety	3	Screening
IES-R	Impact of Event Scale-Revised	PTSD	1	Screening
K-SADS-E	Kiddie-Schedule for Affective Disorders and Schizophrenia (Epidemiological)	Depression	1	Diagnosis
K-SADS-PL	Kiddie-Schedule for Affective Disorders and Schizophrenia (Present & Lifetime Version)	PTSD	4	Diagnosis
MASC	Multidimensional Anxiety Scale for Children	Depression, anxiety	1	Screening
MINI-KID	Mini International Neuropsychiatric Interview for Children and Adolescents	Depression	6	Diagnosis
NOK	Ndetei–Othieno–Kathuku Scale	Depression, anxiety	1	Screening
PC-PTSD	Primary Care PTSD Screen	PTSD	1	Screening
PC-PTSD–5	Primary Care PTSD Screen for DSM–5	PTSD	1	Screening
PDS	Posttraumatic Stress Diagnostic Scale	PTSD	1	Diagnosis
PHQ–9	Patient Health Questionnaire–9	Depression	8	Screening
PSC	Pediatric Symptom Checklist	ADHD, anxiety/depression, conduct problems	1	Screening
RCMAS	Revised Children’s Manifest Anxiety Scale	Anxiety	3	Diagnosis
RQC	Reporting Questionnaire for Children	Conduct problems	1	Screening
SDQ	Strengths & Difficulties Questionnaire	Conduct, ADHD	3	Screening
Short Screening Scale for PTSD	Short Screening Scale for PTSD	PTSD	1	Screening
SMFQ	Short Mood & Feelings Questionnaire	Depression	1	Screening
SNAP-IV	Swanson, Nolan and Pelham Scale	ADHD	1	Screening
SRQ–20	Self-Reporting Questionnaire 20-Item	Depression	1	Screening
TSCC-A	Trauma Symptom Checklist for Children-Alternate Form	PTSD	1	Screening
UCLA PTSDRI	UCLA PTSD Reaction Index	PTSD	4	Screening
VADPRS	Vanderbilt ADHD Diagnostic Parent Rating Scale	ADHD, conduct problems, anxiety/depression	1	Diagnosis
VADTRS	Vanderbilt ADHD Teacher Rating Scale	ADHD, conduct problems, anxiety/depression	1	Diagnosis
YSR	Youth Self Report	Conduct problems, ADHD, anxiety	3	Screening

### Appendix D: Results of Sensitivity Analysis after Removal of Studies with Special Populations and Non-Validated Tools

**Table:**

Selected disorder	Analysis	Prevalence (%)	95%CI (%)	95%PI (%)	*I*^2^ (%)	95%CI (%)
PTSD	Main analysis	12.53	7.59–20.00	1.22–62.41	98.6	98.3–98.9
	Special population removed	14.98	3.59–45.49	0.23–93.16	99.4	99.2–99.5
	Non-validated tools removed	11.31	6.99–17.80	1.32–54.98	98.5	98.2–98.8
	Outliers and influential cases removed	9.74	6.87–13.62	2.99–27.39	89.7	83.6–93.5
	Screening tools removed	8.96	5.21–14.99	2.01–32.05	92.8	86.3–96.3
Depression	Main analysis	15.27	9.92–22.78	0.97–76.80	99.1	99.0–99.2
	Special population removed	13.76	6.17–27.91	0.34–88.07	99.4	99.3–99.5
	Non-validated tools removed	13.56	9.04–19.85	1.15–67.93	99.0	98.9–99.1
	Outliers and influential cases removed	15.14	10.30–21.69	1.63–65.72	97.9	97.5–98.2
	Screening tools removed	7.73	3.54–16.05	0.40–63.63	97.6	96.9–98.2
ADHD	Main analysis	6.55	4.61–9.23	1.20–28.76	98.3	98.0–98.6
	Special population removed	6.58	4.33–9.88	1.18–29.32	98.7	98.5–99.0
	Non-validated tools removed	6.53	4.35–9.69	1.03–31.94	98.5	98.2–98.8
	Outliers and influential cases removed	6.35	5.10–7.87	3.13–12.46	80.8	68.2–88.5
	Screening tools removed	4.82	2.38–33.02	0.52–33.02	93.6	90.0–95.9
Anxiety	Main analysis	11.78	7.27–18.54	1.10–61.61	98.0	97.6–98.4
	Special population removed	10.49	4.39–23.05	0.55–71.43	98.5	98.0–98.8
	Non-validated tools removed	11.44	6.89–18.40	1.00–62.20	98.1	97.6–98.4
	Outliers and influential cases removed	15.19	10.42–21.63	2.70–53.59	97.5	96.8–98.0
	Screening tools removed	10.60	4.54–22.82	0.40–77.69	98.3	97.8–98.6
Conduct disorder	Main analysis	9.76	4.93–18.41	0.79–59.54	97.8	97.1–98.3
	Special population removed	12.07	5.08–26.06	0.92–66.92	98.1	97.3–98.7
	Non-validated tools removed	9.79	4.57–19.72	0.65–64.15	97.9	97.2–98.4
	Outliers and influential cases removed	9.91	4.82–19.28	0.97–55.13	93.9	90.5–96.1
	Screening tools removed	3.43	0.65–16.13	0.02–84.03	95.0	90.1–97.4

Note: This table displays the results of sensitivity analyses that were conducted to assess heterogeneity after the removal of studies that included special population(s) or non-validated tools, outliers and influential cases and screening tools, respectively. Heterogeneity remains high (*I*^2^ > 80%) despite the removal of the above-mentioned studies across all conditions.

### Appendix E: Results of Subgroup Analysis of General vs Special Populations

**Table:**

Selected disorder	Population	Pooled prevalence (%)	95%CI (%)	*I*^2^ (%)	*p*_subgroup_
PTSD	General Special	14.98 11.72	3.59–45.49 6.37–20.57	99.4 97.4	0.66
Depression	General Special	13.76 16.86	6.17–27.91 10.81–25.34	99.4 98.1	0.62
ADHD	General Special	6.58 6.45	4.33–9.88 2.66–14.83	98.7 91.9	0.96
Anxiety	General Special	10.49 12.90	4.39–23.05 6.63–23.61	98.5 97.7	0.66
Conduct disorder	General Special	12.07 7.22	5.08–26.06 1.39–30.12	98.1 95.8	0.43

Note: No significant differences were observed between general and special populations for all conditions.

### Appendix F: Results of Subgroup Analysis of Validated vs Non-Validated Tools

**Table:**

Selected disorder	Validation of tools	Pooled prevalence (%)	95%CI (%)	*I*^2^ (%)	*p*_subgroup_
PTSD	Validated Not validated*	11.31 53.77	6.99–17.80 48.15–59.30	98.5 –	< 0.0001
Depression	Validated Not validated	13.56 65.58	9.04–19.85 0.00–100.00	99.0 99.3	0.11
ADHD	Validated Not validated	6.53 7.07	4.35–9.69 3.21–14.86	98.5 52.9	0.76
Anxiety	Validated Not validated*	11.44 20.59	6.89–18.40 18.32–23.07	98.1 –	0.01
Conduct disorder	Validated Not validated*	9.79 9.26	4.57–19.72 7.69–11.11	97.9 –	0.87

Note: Significant differences were observed between studies, including validated compared to non-validated tools for PTSD and anxiety. In both cases, studies using non-validated tools were disproportionately less and had a higher reported pooled prevalence than those using validated tools.

* *n* = 1

### Appendix G: Results of Subgroup Analysis of Screening vs Diagnostic Tools

**Table:**

Selected disorder	Population	Pooled prevalence (%)	95%CI (%)	*I*^2^ (%)	*p*_subgroup_
PTSD	Screening Diagnostic	13.90 8.96	7.05–25.56 5.21–14.99	98.6 92.8	0.23
Depression	Screening Diagnostic	22.00 7.73	13.67–33.43 3.54–16.05	99.3 97.6	0.01
ADHD	Screening Diagnostic	7.94 4.82	5.26–11.82 2.38–9.51	98.8 93.6	0.16
Anxiety	Screening Diagnostic	13.34 10.60	8.64–20.04 4.54–22.82	96.8 98.3	0.58
Conduct disorder	Screening Diagnostic	15.68 3.43	8.92–26.09 0.65–16.13	97.7 95.0	0.01

Note: Significant differences were observed for depression and conduct disorder, where the pooled prevalence of studies using a diagnostic tool was lower than studies using a screening tool.
