# Supplementary material for: Meta-Analysis: Prevalence of Youth Mental Disorders in Sub-Saharan Africa
Source: Glob Ment Health (Camb). 2024 Nov 14;11:e109. doi: 10.1017/gmh.2024.82 (PMC11704384; doi:10.1017/gmh.2024.82)
Supplement: Jakobsson et al. supplementary material [file S2054425124000827sup001.docx]

**Supplement 1. Prospero Registration**

XXX XXX, XXX XXX, XXX XXX, XXX XXX, XXX XXX. Prevalence of Youth Mental Disorders

in sub-Saharan Africa: A Meta-Analysis . PROSPERO 2022 XXXX

Review question

What is the prevalence of common mental disorders among youth in sub-Saharan Africa? Are certain youth demographic groups disproportionately affected by mental disorders? This study aims to expand upon a meta-analysis performed by Cortina et.al. (2012) that gathered prevalence data for general psychological difficulties in children living in sub-Saharan Africa by detailing the prevalence of specific mental disorders by making use of studies performed in the years since 2012. Through this meta-analysis, we hope to also explore the effects of several moderators of prevalence (i.e screening questionnaires vs. clinical diagnostics, gender, age, location, etc.).

Searches

We developed search protocols for 4 databases: Africa-wide information, PsycINFO, PubMed, and Africa Journal Online. Using these 4 databases, we searched for prevalence studies across all sub-Saharan African nations. We limited our results to those published in English. Due to limitations in the African Journals Online user-interface, only the first 100 articles (sorted by relevance) could be included. Finally, we have found and will continue to find more articles by

following reference trails and recommendations from researchers in the field of youth mental health. For this study, we have limited our searches to articles in English that were published 1960 and after.

Types of study to be included

This meta-analysis will include published, peer-reviewed studies of the prevalence of mental disorder(s) among youth in SSA. Included studies must report epidemiological survey data on mental illness falling under one of the five domains of interest, and the population surveyed must be of the general population or of a special population not defined by the presence of mental illness (i.e., not those who sought treatment for mental illness at a clinic, but a population of youth

with HIV).

Condition or domain being studied

We will include prevalence studies targeting five domains of youth psychopathology: depression problems, anxiety, posttraumatic stress disorder, conduct problems, and ADHD problems.

Participants/population

Age range (mean age: 0-18.4) including studies not specific to this age range but with results for this age range reported separately; published in English; reports mental disorder prevalence using a measure of mental health diagnostic status or an established (i.e., psychometrically validated in some setting) symptom levels disorder types (anxiety problems, depression problems, conduct problems, ADHD problems, post-traumatic stress); the study is of the general population and special populations that are not already selected or self-selected for mental health problems or symptoms (i.e., the study cannot be of a group of people already seeking mental health services);

the study occurred in one or more countries in sub-Saharan Africa (i.e., not a refugee or migrant population in another country).

Intervention(s), exposure(s)

Not Applicable.

Comparator(s)/control

Not Applicable.

Context

This meta-analysis includes studies of youths (mean age: 0-18.4) living in SSA. As the geographical context is an important factor, studies involving youths from SSA living in another country (e.g. migrants or refugees etc) will not be included in this review. Additionally, we will exclude studies that include youths already seeking help for mental health problems.

Main outcome(s)

Prevalence of several common mental disorders across all youth samples in sub-Saharan Africa.

Measures of effect

Prevalence measure often expressed as a percentage.

Additional outcome(s)

Differences in prevalence of specific mental disorders across various demographic breakdowns (age, gender, country, etc).

Measures of effect

Prevalence measure often expressed as a percentage.

Data extraction (selection and coding)

Studies will be identified via the three search procedures described: First, we will search PubMed and PsycINFO for relevant studies, second we will search the international databases with their respective search strategies, and third we will follow the reference trails of relevant works and recommendations from researchers in the field of global youth mental health. Studies will be screened based on the inclusion and exclusion criteria noted previously. The data to be extracted will include study characteristics, diagnostics tools, and sample characteristics. Data extraction will be conducted by four researchers (CJ, NJ, RB and BO) and discrepancies will be resolved through discussion.

Risk of bias (quality) assessment

Study quality will be assessed using the Joanna Briggs Institute critical assessment tools.

Strategy for data synthesis

To investigate the primary research question, we will use the statistical software R to conduct robust variance estimation (RVE).

Analysis of subgroups or subsets

We plan to investigate the differences in the prevalence of specific mental disorders across various demographic breakdowns (age, gender, country, etc).

Contact details for further information

XX XX

XXX@XX.XX

Organisational affiliation of the review

XX XX

https://www.xx.xx/

Review team members and their organisational affiliations

XX XX. XX XX and XX XXX XXX

XX XX. XX XX

XX XX. XX XX

XX XX. XX XX

XX XX. XX XX and XX XX

Type and method of review

Diagnostic, Epidemiologic, Meta-analysis, Systematic review

Anticipated or actual start date

20 December 2021

Anticipated completion date

15 June 2022

Funding sources/sponsors

None

Grant number(s)

State the funder, grant or award number and the date of award

None

Conflicts of interest

Language

English

Country

England, Kenya, United States of America

Stage of review

Review Ongoing

Subject index terms status

Subject indexing assigned by CRD

Subject index terms

Adolescent; Africa South of the Sahara; Humans; Mental Disorders; Prevalence

Date of registration in PROSPERO

10 May 2022

Date of first submission

19 April 2022

Details of any existing review of the same topic by the same authors

Our team has published several previous meta-analyses, but no one on our team has published a meta-analysis like the one described here.

Stage of review at time of this submission

**Stage Started Completed**

Preliminary searches Yes Yes

Piloting of the study selection process Yes Yes

Formal screening of search results against eligibility criteria Yes No

Data extraction No No

Risk of bias (quality) assessment No No

Data analysis No No

*The record owner confirms that the information they have supplied for this submission is accurate and complete and they understand that deliberate provision of inaccurate information or omission of data may be construed as scientific misconduct. The record owner confirms that they will update the status of the review when it is completed and will add publication details in due course.*

Versions

10 May 2022

10 May 2022

Powered by

**Supplement 2. Prisma Flow Chart for Included Studies**

**
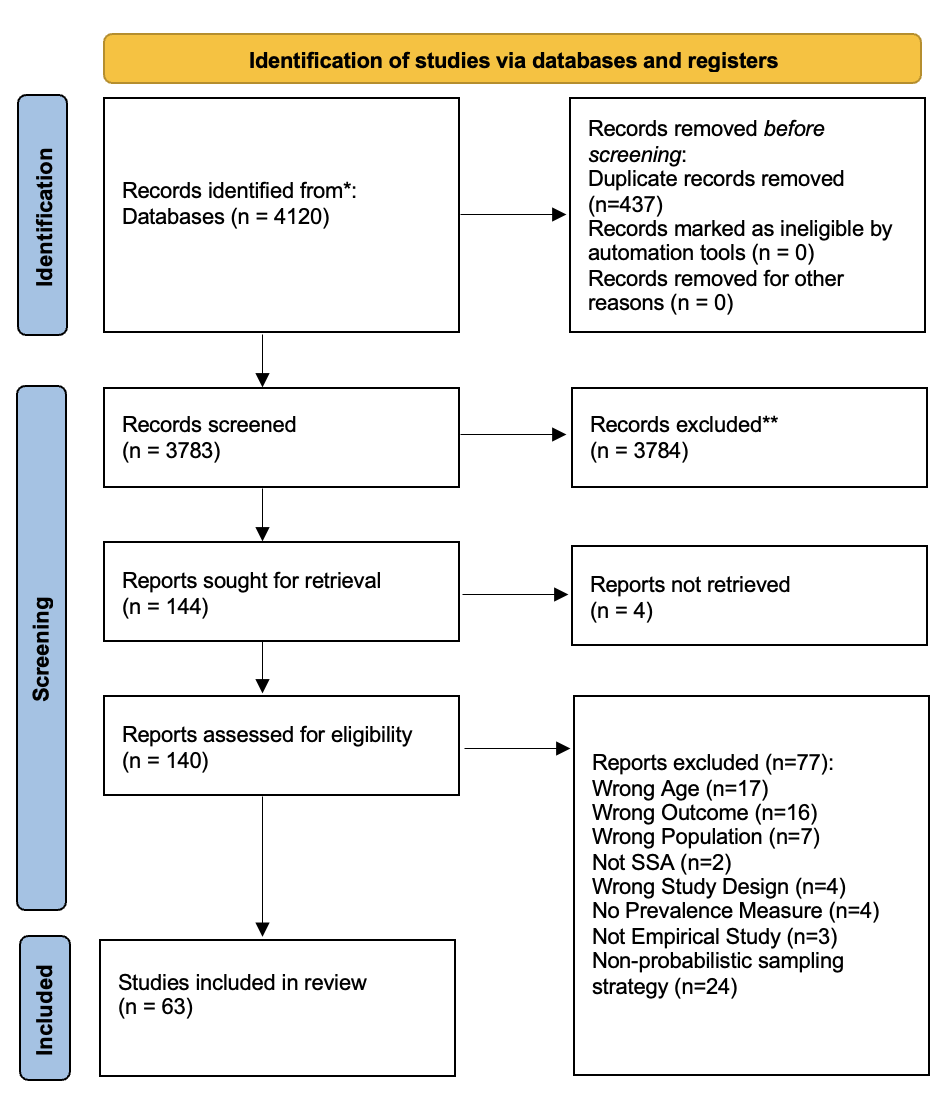
**

**Supplement 3. Disorder Specific Forest Plots**

*Post-Traumatic Stress Disorder Forest Plot*

*
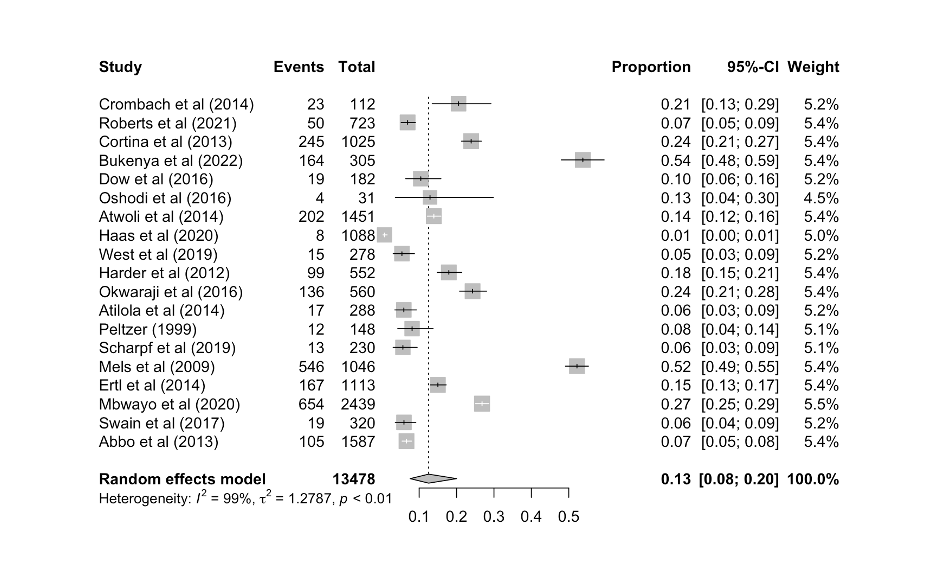
*

*Depression Forest Plot*

*
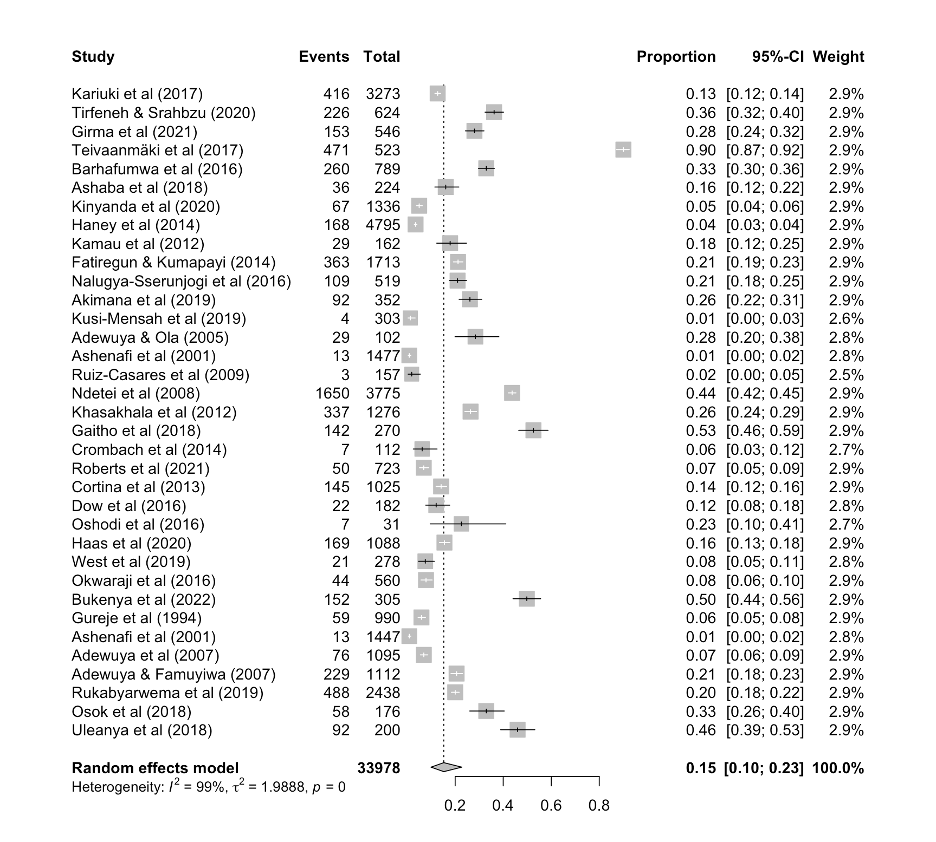
*

*Attention Deficit Hyperactivity Disorder Forest Plot*

*
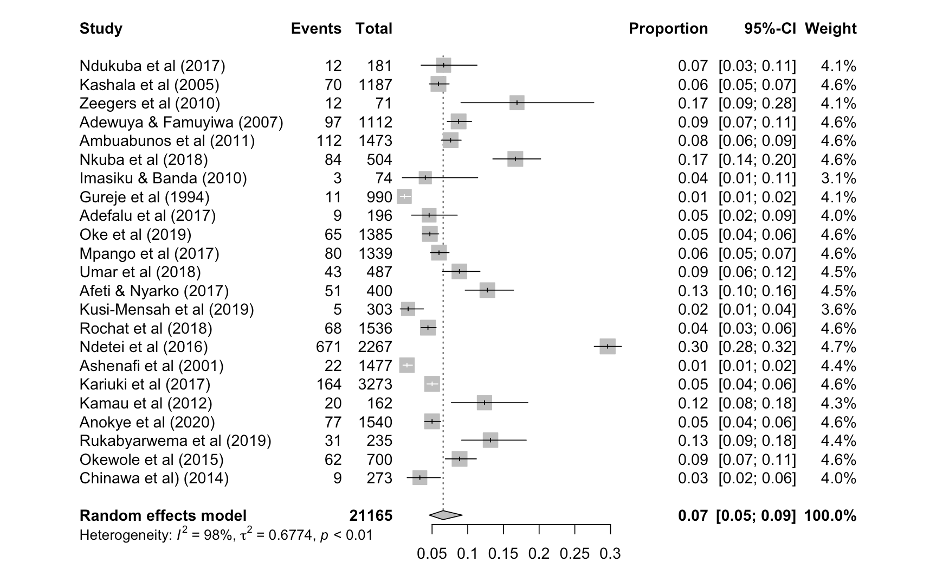
*

*Anxiety Disorder Forest Plot*

*
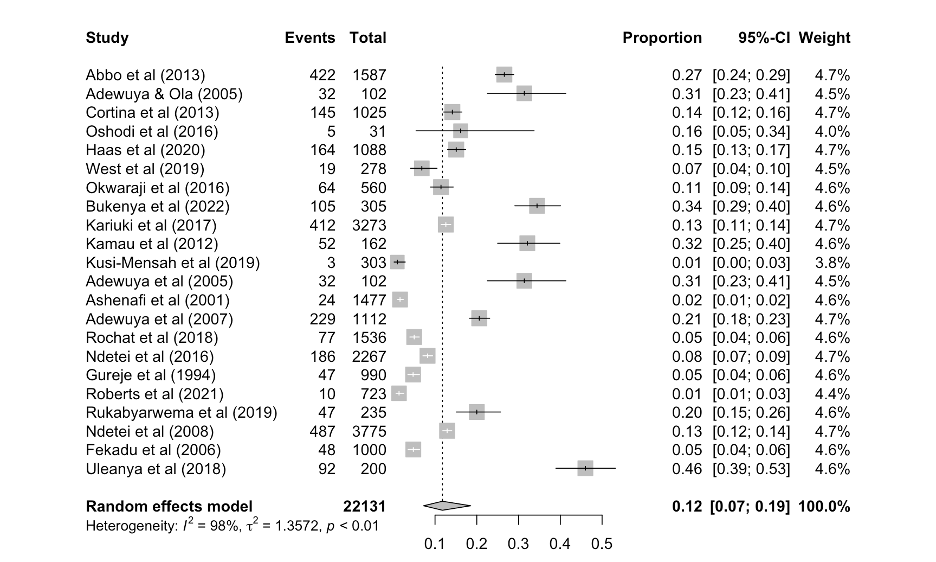
*

*Conduct Problems Forest Plot*

*
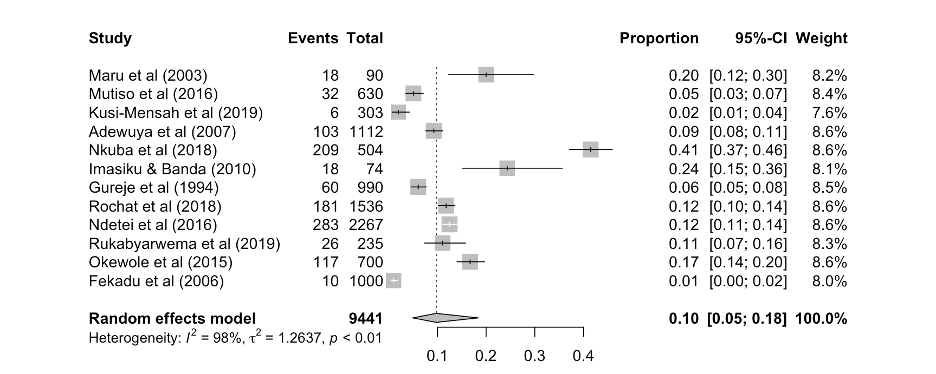
*
