# Supplementary material for: Meta-Analysis: Prevalence of Youth Mental Disorders in Sub-Saharan Africa
Source: Glob Ment Health (Camb). 2024 Nov 14;11:e109. doi: 10.1017/gmh.2024.82 (PMC11704384; doi:10.1017/gmh.2024.82)
Supplement: Peer review history

# Editor report: Recommendation: Meta-Analysis: Prevalence of Youth Mental Disorders in Sub-Saharan Africa — R0/PR2

**DOI:** 10.1017/gmh.2024.82.pr2

**Affiliations:** Psychiatry, Makerere University CHS, Uganda

Dear Authors,

In addition to addressing the comments from the reviews, please consider including studies published beyond 2021 when your search was carried out, at least in the discussion section as this would improve your paper fitting in the global published research for the disorders discussion, particularly PTSD. Below are some references:

Yang F, Wen J, Huang N, Riem MME, Lodder P, Guo J. Prevalence and related factors of child posttraumatic stress disorder during COVID-19 pandemic: A systematic review and meta-analysis. Eur Psychiatry. 2022 Jun 21;65(1):e37. doi: 10.1192/j.eurpsy.2022.31. PMID: 35726735; PMCID: PMC9280924.

Woolgar, Francesca, Harriet Garfield, Tim Dalgleish, and Richard Meiser-Stedman. 2022. “Systematic Review and Meta-analysis: Prevalence of Posttraumatic Stress Disorder in Trauma-Exposed Preschool-Aged Children.” Journal of the American Academy of Child & Adolescent Psychiatry 61 (3): 366-377. https://doi.org/https://doi.org/10.1016/j.jaac.2021.05.026. https://www.sciencedirect.com/science/article/pii/S0890856721004238

# Editor report: Decision: Meta-Analysis: Prevalence of Youth Mental Disorders in Sub-Saharan Africa — R0/PR3

**DOI:** 10.1017/gmh.2024.82.pr3

**Affiliations:** London School of Hygiene & Tropical Medicine, United Kingdom of Great Britain and Northern Ireland

No accompanying comment.

# Editor report: Recommendation: Meta-Analysis: Prevalence of Youth Mental Disorders in Sub-Saharan Africa — R1/PR5

**DOI:** 10.1017/gmh.2024.82.pr5

**Affiliations:** Psychiatry, Makerere University CHS, Uganda

No accompanying comment.

# Editor report: Decision: Meta-Analysis: Prevalence of Youth Mental Disorders in Sub-Saharan Africa — R1/PR6

**DOI:** 10.1017/gmh.2024.82.pr6

**Affiliations:** London School of Hygiene & Tropical Medicine, United Kingdom of Great Britain and Northern Ireland

No accompanying comment.

# Editor report: Recommendation: Meta-Analysis: Prevalence of Youth Mental Disorders in Sub-Saharan Africa — R2/PR8

**DOI:** 10.1017/gmh.2024.82.pr8

**Affiliations:** Psychiatry, Makerere University CHS, Uganda

No accompanying comment.

# Editor report: Decision: Meta-Analysis: Prevalence of Youth Mental Disorders in Sub-Saharan Africa — R2/PR9

**DOI:** 10.1017/gmh.2024.82.pr9

**Affiliations:** London School of Hygiene & Tropical Medicine, United Kingdom of Great Britain and Northern Ireland

No accompanying comment.
